# Novel Iron Chelator SK4 Drives Cytotoxicity through Inhibiting Mitochondrial Metabolism in Ovarian and Triple Negative Breast Cancer Cell Lines

**DOI:** 10.3390/biomedicines11072073

**Published:** 2023-07-24

**Authors:** Gina Abdelaal, Andrew Carter, William Cheung, Mihalis Panayiotidis, Seth Racey, David Tétard, Stephany Veuger

**Affiliations:** 1 Department of Applied Sciences, Faculty of Health and Life Sciences, Northumbria University, Newcastle upon Tyne NE1 8ST, UK; ginaamr96@gmail.com (G.A.); andrew.carter@northumbria.ac.uk (A.C.); william.cheung@northumbria.ac.uk (W.C.); david.tetard@northumbria.ac.uk (D.T.); 2 Department of Cancer Genetics, Therapeutics & Ultrastructural Pathology, The Cyprus Institute of Neurology & Genetics, Nicosia 2371, Cyprus; mihalisp@cing.ac.cy

**Keywords:** iron, cancer, mitochondria, iron chelator, therapy, SK4

## Abstract

Anti-cancer therapy by iron chelation has been shown to inhibit many cellular processes including DNA replication, mitochondrial metabolism and oncogenic signalling pathways (e.g., EGFR). Iron chelator SK4 represents a double pronged approach towards treating cancer. SK4 enters through LAT1, a commonly overexpressed amino acid transporter in tumours, thus targeting iron addiction and LAT1 overexpression. The aim of this study was to characterise the mode of action of SK4 through proteomics, metabolomics, lipidomics and seahorse real-time analysis in ovarian cell line SKOV3 and triple negative breast cancer cell line MDA MB 231. Pathway enrichment of proteomics data showed an overrepresentation of metabolism related pathways. Metabolic change after SK4 exposure have been confirmed in investigations of changes in basal and maximal mitochondrial respiration using seahorse real-time analysis of mitochondrial metabolism. Metabolomics also showed an increase in AMP and glucose-1-phosphate. Interestingly, our lipidomics data show a decrease in phospholipid synthesis in the SKOV3 cells which is in contrast with previous data which showed an upregulation of ceramide driven apoptosis. In summary, our data highlight impairment of energy metabolism as a mechanism of action underlying SK4 apoptosis, but also suggest a potential role of ceramide induction in the phenotypic outcome of the cell model.

## 1. Introduction

Iron chelators are a class of anti-cancer therapy which target the dependency of cancer on iron for growth and proliferation [1]. Several mechanisms have been proposed to underly iron chelator cytotoxicity. Iron chelation blocks cell cycle progression through stimulating and inhibiting the expression or function of cell cycle related proteins (e.g., ribonucleotide reductase which is the rate limiting step of DNA synthesis) [2]. Iron chelation has been shown to induce many forms of cell death including apoptosis and autophagy [1,3]. Another proposed target of iron chelation is NDRG1, a tumour suppressor capable of inhibiting EGFR, wnt/β-catenin and STAT3 signalling [4,5,6]. Overall, the clear pattern is multiple targets that could potentially be working in concert to induce cytotoxicity. Although iron chelation has been shown to be effective when tested in vitro and in vivo, this success has not been achieved in clinical trials. The iron chelator Triapine, for example, can induce BRCAness in BRCA wildtype cells and is a more potent inhibitor of ribonucleotide reductase than hydroxyurea [7,8]. However, when taken forward for clinical trials, Triapine had a short-lived patient response indicating potential resistance [9]. This highlights the necessity of designing novel iron chelators with improved targeting of cancer cells.

SK4 is a novel iron chelator designed and synthesised within our group. SK4 is composed of two key moieties; a moiety responsible for iron chelation and a moiety responsible for entry through LAT1—an amino acid transporter which is commonly overexpressed in cancer cells [10]. Initial work within our group showed that SK4 drove a loss of cell viability and subsequent apoptosis in an in vitro model for melanoma which was found to be dependent on ceramides [10]. This led to further investigation utilising a more extensive panel of cell lines which demonstrated SK4 was effective against a range of cell lines including p53 mutant cell lines (e.g., prostate PC3 cells) and in triple negative breast cancer cell lines (e.g., breast MDA MB 231 cells). Interestingly, a differential response to SK4 was observed in breast MDA MB 231 cells compared to ovarian SKOV3 cells. The MDA MB 231 cell line underwent apoptosis, whereas the SKOV3 cell line did not and, whilst both cell lines displayed S phase arrest, it remained until 72 h in the MDA MB 231 cells but reverted to normal cell cycle distribution at 48 h in the SKOV3 cells [11].

The main aim of this paper was to further analyse the mode of action of SK4 using a multiomics approach of proteomics, metabolomics and lipidomics to establish a clearer understanding of the impact of iron chelation on cellular pathways and potential limitations. Iron is known to be a cofactor of many crucial enzymes including ribonucleotide reductase and iron can influence gene expression through binding to IRP1/2. Additionally, the metallome is poorly characterised so iron may also have some unknown functions within cancer cells; thus, we hypothesise iron chelation treatment could have many targets.

## 2. Materials and Methods

### 2.1. Cell Culture and Treatments

MDA MB 231 (Sigma Aldrich, St. Louis, MO, USA) and SKOV3 (Sigma Aldrich) cell lines were routinely passaged in DMEM (Gibco, Waltham, MA, USA) and Mcoy’s media (Gibco, Waltham, MA, USA) supplemented with 10% FBS (Gibco, Waltham, MA, USA) and 1% penicillin/streptomycin (Gibco, Waltham, MA, USA). Cells were maintained at 37 °C and 5% carbon dioxide in a humidified incubator. Passages were limited to 20 subsequent passages and cells were subjected to routine mycoplasma testing. For further experiments, cells were treated with media supplemented with SK4 at the appropriate concentrations which were established through previous work [11].

### 2.2. Proteomics

Cells were grown in 6-well plates at a cell density of 2 × 10^5^/mL and treated with SK4 for 24 h on the following day. SKOV3 cells were treated with 146 μM of SK4, and MDA MB 231 cell were treated with 316 μM for 24 h. Cells were scraped off in 1× PBS, two technical replicates were pooled together for each condition and spun down at 3000 RPM for 3 min. All reagents used for proteomics were LC-MS grade. Pellets were resuspended in 50 μL of 5% SDS, 50 mM TEAB pH 8.5 buffer and left on ice for 30 min. Cell lysates were sonicated using a water bath sonicator on ice for 5 min. Lysates were spun down at 12,000 *g* for 10 min, and then, frozen at −20 °C until ready. A total of at least 4 biological replicates were collected. Lysates were assessed for their protein content using BCA assays and samples of protein lysates were adjusted to 50 μg, and then, reductant TCEP was added at 5 mM, and then, lysates were incubated at 55 °C for 15 min. Lysates were spun down briefly and incubated with 20 mM of the alkylator MMTS at room temperature for 10 min. To acidify the lysates, 2.5% of phosphoric acid was added and the lysates were vortexed. A volume of 165 μL binding buffer was added to the lysates, mixed through pipetting up and down and transferred to an S-trap^TM^ spin column. The spin columns were centrifuged at 4000× *g* for 30 s to ensure protein trapping. The spin columns were washed 3 times in 150 μL binding buffer 4000 *g* for 30 s. For trypsin digestion, 2 μg of trypsin was added to each spin column and incubated in a 47 °C water bath for 2 h. The peptides were eluted in three consecutive 4000 *g* 1-min spins in 40 μL of 50 mM TEAB, followed by 40 μL of 0.2% formic acid, and finally, 40 μL 50% acetonitrile. Elutions were handed to the Newcastle proteomics facility for further processing and mass spectrometry. Samples were injected onto an RSLC nano HPLC system where they are separated according to their hydrophobicity on a c18 column. Peptides are elutedonline into a Thermo Exploris 480 mass spec for tandem MS or Data Independent Acquisition (DIA). Following the return of the list of identified peptides from the proteomics unit, data were analysed using Perseus for log2 transformation and R package Limma for differential expression [12,13]. Pathway enrichment was performed through overrepresentation analysis using ReactomePA [14].

### 2.3. Metabolomics and Lipidomics Sample Preparation

Cells were seeded at 3 × 10^6^ in T75 flasks. Following 24 h, media were removed and replaced with fresh media or media spiked with drug. The SKOV3 cells were treated with 146 μM of SK4, and MDA MB 231 cell were treated with 316 μM for 24 h, and then, harvest in 1X PBS and spun down at 3000 RPM for 3 min. Cells were counted as previously described and 3 × 10^6^ were collected. Cell pellets were washed 3 times in 1X PBS at 3000 RPM for 3 min. The samples were extracted using a Folch biphasic extraction methodology. The cell pellet was immersed in 1 mL of 2:1 methanol/chloroform and vortex for 30 s and then sonicated in a water ice bath for 15 min. After, the 500 μL of LC/MS grade water was added to the extraction buffer and vortexed for 30 s and sonicated in ice water bath for additional 10 min to induce phase separation of the metabolite and lipid component. The extraction samples were then centrifuged for 5000 RPM at 10 °C for 15 min. The upper aqueous layer containing the metabolite component was collected and dried down in a SpeedVac at 45 °C for 2 h or until all extraction buffer was removed. The same was applied for the organic lower layer containing the lipids. Both fractions were preconcentrated independently and stored at −20 °C afterward until analysis.

### 2.4. Mass Spectrometry Acquisition Metabolomics

The metabolite fraction was reconstituted in 100 μL of 95/5 *v*/*v* ACN/LC/MS grade water and sonicated for 15 min. The samples were centrifuged at 15, 000 RPM for 15 min at 4 °C and filtered via 0.22 micro Costar at 5 K rpm and transferred to an autosampler with 200 uL microinsert and capped. The remaining samples were used to create a pooled QC for data acquisition and for the acquireX DDA workflow. Extraction blanks were also incorporated as part of the sample analysis workflow. Metabolite and lipid characterisation of the cell line were performed on a Thermo Scientific (Hemel Hempstead, UK) Vanquish Liquid chromatography Front end connected to IDX High Resolution Mass Spectrometer system. The MS data were acquired using the AcquieX acquisition workflow (data dependent analysis). The MS was operating parameters were as follows: MS1 mass resolution 60 K, for MS2 30 K stepped energy (HCD) 20, 25, 50 scan range 100–1000, RF len (%) 35, AGC gain, intensity threshold 2e4 25% custom injection mode with an injection time of 54 ms. An extraction blank was used to create background exclusion list and a pooled QC was used to create the inclusion list. Through Hydrophilic Liquid Interaction Chromatography (HILIC), the chromatographic separation was archived using a Waters Acquity UPLC BEH amide column (2.1 × 150 mm with particle size of 1.7 μm), part no 186004802, operating at 45 °C with a flow rate of 200 μL/min. The LC gradient consist of binary buffer system, buffer A (LC/MS grade water) and Buffer B (LC/MS ACN) both containing with 10 mM ammonium formate. For positive mode, the pH of buffers was adjusted using 0.1% formic acid. The LC gradients were the same for both polarity—95% B at T0 hold for 1.5 min and linearly decrease to 50%B at 11 min hold for 4 min and return to starting condition and hold for further 4.5 min (column stabilization). The voltage applied for positive mode and negative mode was 3.5 kV. Injection volume used: positive mode 3 μL.

### 2.5. Mass Spectrometry Acquisition Lipidomics

The Lipid fraction was reconstituted in 100 μL of 1:1:2 (water/ACN/IPA); samples were sonicated for 15 min and centrifuged for 15 min at 15,000 RPM before transferring 80 μL to autosampler vial with 200 μL microinsert. The remaining samples were used to create a pooled QC for data acquisition and for the acquireX DDA workflow. Extraction blanks were also incorporated as part of the sample analysis workflow. Lipidomics: The MS data were acquired using the acquireX acquisition worflow (data dependent analysis). The MS operating parameters were as follows: MS1 mass resolution 60 K, MS2 30 K scan range 250–1250, stepped energy (HCD) 20,25,50. RF len (%) 35, AGC gain, intensity threshold 2e4 25% custom injection mode with an injection time of 54 ms. The chromatographic separation was archived using Waters Acquity UPLC CSH C18 column (2.1 × 150 mm with particle size of 1.7 μm), part no 186005298, operating at 55 °C with flow rate if 200 μL/min. The LC gradient consist of binary buffer system, buffer A (LC/MS grade water/ACN 40/60 *v*/*v*), Buffer B (90:10 IPA and ACN), with both buffers containing 10 mM ammonium formate. Independent buffer systems were used for positive and negative mode acquisition, respectively, for positive mode; the pH of buffers was adjusted using 0.1% formic acid and for negative 0.1% Ammonia solution. The LC gradients were the same for both polarity: 60% B at T0 hold for 1.5 min and linearly increase to 85% B at 7 min and then increased to 95% B at 12.5 min and hold for 4.5 min return to starting condition and hold for further 4.5 min (column stabilization). Injection volume used positive mode with 3 L and negative mode 5 μL, respectively. The voltage applied for positive mode and negative mode was 3.5 kV and 2.5 kV, respectively. The HESI conditions were as follows for 200 μL: Sheath Gas: 35, Aux Gas 7 and c Sweep Gas of 0. Ion Transfer tube Temp: 300 °C and Vaporizer Temp 275 °C. The HESI Conditions were the same for HILIC and Lipidomics. Post data processing: The HILIC Pos and Neg data sets were processed via Compound Discoverer 3.3 according to the following sittings: Untargeted metabolomic workflow: mass tolerance 10 ppm, maximum shift 0.3 min, alignment model adaptive curve, minimum intensity 1e6, S/N threshold 3, compound consolidation, mass tolerance 10 ppm, RT tolerance 0.3 min. Database matching were performed at MS2 level using Thermo scientific *m*/*z* cloud with a similar index of 70% or better. The lipidomic Pos and Neg data set were processed via Thermo scientific Lipid search version 4 workflow: HCD (high energy collision database) retention time (min) 0.1 min, parent ion mass tolerance (5 ppm), product ion (10 ppm). Alignment method (max), top rank off, Minimum m-score 5.0, all isomer peaks, ID quality filter A and B only. Lipid ID were matched using lipid search in silico library MS2 level. Quality control: Corresponding HILIC and Lipidiomics pooled QCs samples were used to assessed for instrumental drifts, The RSD variation across the QCs for Hilic and Lipidomics were less than 15% respectively. Any metabolite/lipid features which has an RSD of 25% or less within the QCs where retained, this is extended to the rest of the data set. The respective data sets were log2 normalized and auto-centred prior to unsupervised analysis using PCA for assessment of global trends and outliners prior to supervised analysis (PLD-DA) for identification of the most discriminate metabolite/lipids. The data visualization and analysis were performed in Metaboabalyst.

### 2.6. Seahorse Real-Time Analysis

Real-time measurements of Oxygen Consumption Rate (OCR) and Extracellular Acidification Rate (ECAR) were performed using a Seahorse XFp Extracellular Flux Analyser (Agilent Technologies, Santa Clara, CA, USA). SKOV3 (a human ovarian cancer cell line) and the MDA MB 231 (an epithelial, human breast cancer cell line) were incubated for 24 h with SK4. The cells were then washed with PBS and dissociated with Trypsin and resuspended in complete media and seeded in a monolayer at 4 × 10^5^ cells per well of an 8-well XFp plate. Cells were incubated at 37 °C in a 5% CO_2_ atmosphere for 24 h. Then, 1 h before analysis, cells were incubated at 37 °C without CO_2_ in the seahorse XF DMEM medium (Agilent Technologies, Santa Clara, CA, USA), supplemented with 10 mM glucose, 2 mM glutamine and 1 mM pyruvate, with or without the IC50 doses of SK4. The Seahorse Mito stress test (Agilent Biosciences, Santa Clara, CA, USA) drugs were injected sequentially (Oligomycin 1.5 M, FCCP 0.5 μM, rotenone/antimycin 0.5 μM) and real-time measurements of OCR and ECAR captured 3 times over a 5 min period following a 3 min mixing period, for 120 min. Each experiment included three repeats per SK4 treatment of the two cell lines and each experiment was repeated three times. The basal respiratory capacity and maximal respiratory capacity were calculated in accordance with Agilent bioscience instructions using their proprietary Wave software (version 2.6). Representative data are shown from one of the three experimental repeats. The Seahorse XFp results were analysed using Agilent’s Wave software and outputs analysed in GraphPad prism using a two-tailed Student’s *t*-test and presented as a mean and standard deviation (error bars). *p* values below *p* < 0.05 (*) and *p* < 0.01 (**) were established as significant. 

## 3. Results

### 3.1. Proteomics Analysis Demonstrates That the MDA MB 231 Cells Are More Significantly Affected by SK4 Treatment Than SKOV3 Cells

**Figure 1:**
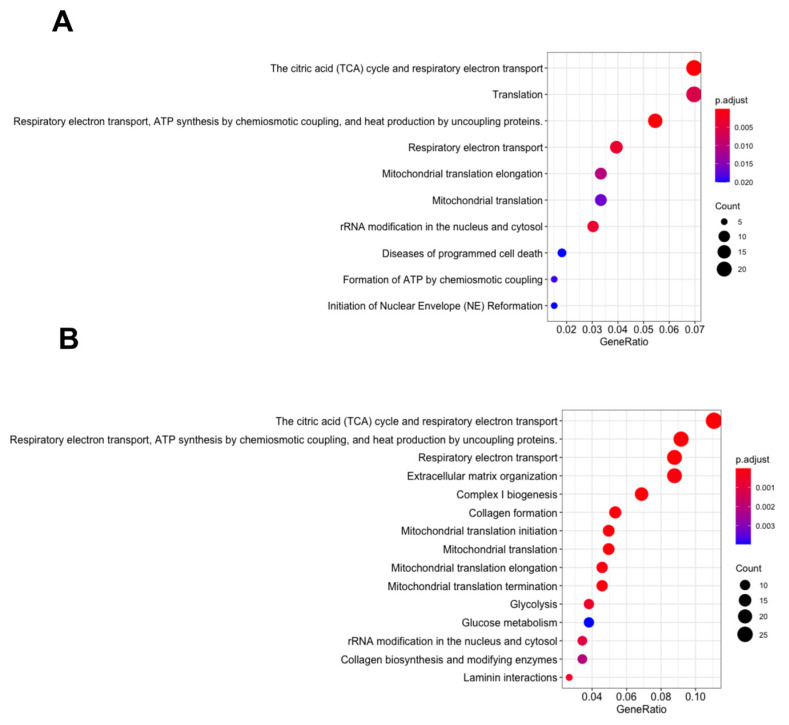
Pathway enrichment for SKOV3 and MDA MB 231 cells treated with SK4. SKOV3 (**A**) and MDA MB 231 (**B**) cells were treated with SK4 for 24 h; proteomic changes were analysed through differential expression using limma and pathway enrichment using Reactome PA.

Previous work within our group demonstrated that, although SK4 decreased cell viability in both the SKOV3 and MDA MB 231 cell lines, there remained a differential response in terms of apoptosis and cell cycle arrest. SKOV3 cells did not display apoptosis and cell cycle analysis showed S phase arrest at 24 h which returned to normal cell cycle distribution at 48 h. MDA MB 231 cells displayed apoptosis and S phase arrest which was retained at 72 h. To determine if the differences in response were due to differences in pathway induction or repression, proteomics analysis was utilised. MDA MB 231 and SKOV3 cells were treated with their appropriate dosage of SK4 (MDA MB 231 = 31 μM, SKOV3 = 146 μM). Differential expression of treated versus untreated cells yielded a list of 372 differentially expressed proteins in the MDA MB 231 cell line (*p* < 0.05) and 31 proteins with an adjusted *p* value < 0.05 (see Table S1). In the SKOV3 cell line, 458 proteins were differentially expressed (*p* < 0.05) with no proteins displaying an adjusted *p* value < 0.05 (See Table S2). Further analysis on the lists of differentially expressed proteins was conducted through pathway enrichment with Reactome PA. Interestingly, pathway enrichment revealed that, although there were some pathway terms unique to the MDA MB 231 breast cancer cells (R-HSA-1474290 Collagen formation, R-HSA-1474244 Extracellular matrix organization) and SKOV3 ovarian cancer cells (R-HSA-445144 Signal transduction by L1), there remained a pattern of mitochondrial pathways which were enriched in both cell lines: R-HSA-1428517 The citric acid (TCA cycle) and respiratory electron transport; R-HSA-163200 Respiratory electron transport, ATP synthesis by chemiosmotic coupling and heat production by uncoupling proteins; and R-HSA-611105 Respiratory electron transport (see Figure 1).

### 3.2. Energy Metabolism Related Pathways Were Enriched in the MDA MB 231 and SKOV3 Cells

**Figure 2:**
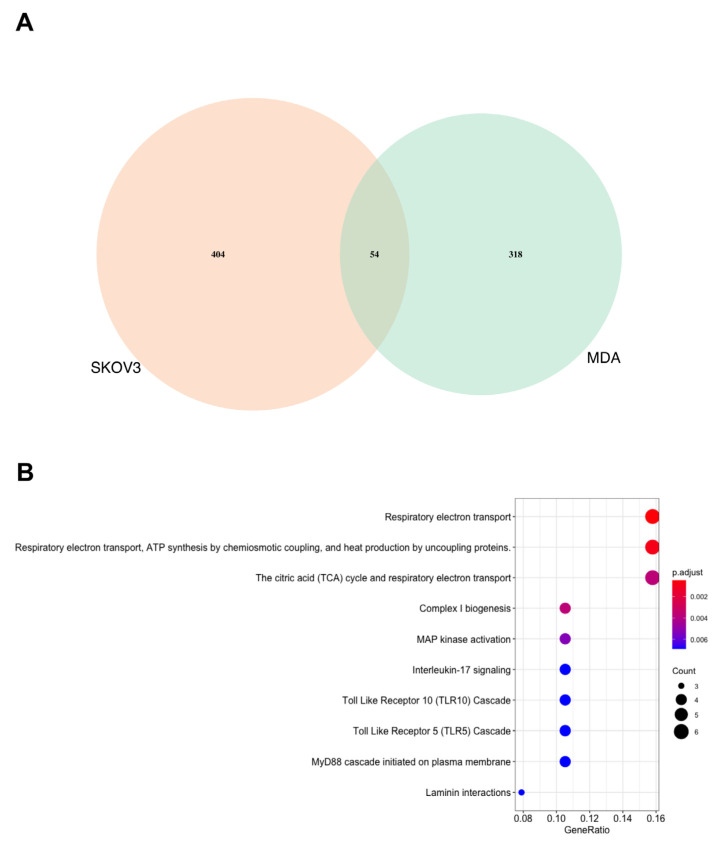
Energy metabolism is perturbed in both SKOV3 and MDA MB 231 cells treated with SK4. (**A**) Venn diagram of differentially expressed proteins in SKOV3 and MDA MB 231 cells treated with SK4. (**B**) dot plot of overrepresented pathways generated through Reactome PA.

To compare the observed SK4 mediated changes in the proteome, a Venn diagram was utilized to contrast the lists of differentially expressed proteins in the MDA MB 231 cells and the SKOV3 cells (*p* < 0.05). There were 54 commonly differentially expressed proteins between the SKOV3 and MDA MB 231 cell lines (see Table S3), as well as 404 proteins uniquely differentially expressed (DE) E in the SKOV3 cells and 318 proteins DE in the MDA MB 231 cells. Pathway enrichment revealed energy metabolism related pathways were enriched among the 54 commonly differentially expressed proteins including R-HSA-1428517 The citric acid (TCA cycle) and respiratory electron transport; R-HSA-163200 Respiratory electron transport, ATP synthesis by chemiosmotic coupling and heat production by uncoupling proteins; and R-HSA-611105 Respiratory electron transport (see Figure 2).

### 3.3. SK4 Upregulates AMP and Glucose-1-Phosphate in SKOV3 Cells

**Figure 3:**
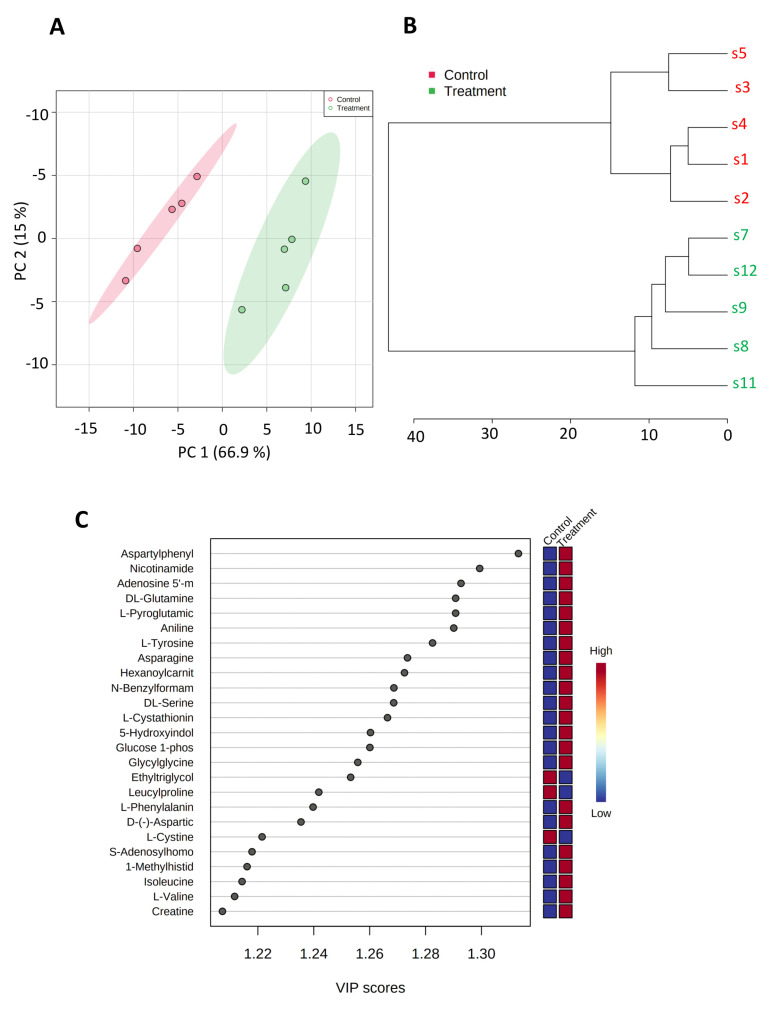
SK4 induces an increase in energy metabolites and amino acid substrates of LAT1 in SKOV3 cells. The metabolomic changes in SKOV3 cells treated with SK4 for 24 h. Quality control of metabolomic data was conducted through PCA (**A**) and HCA (**B**) analysis. Data are representative of five biological replicates. (**C**) dot plot of top 25 perturbed metabolites VIP score > 1 generated through PLS-DA.

To further assess the impact of SK4 on energy metabolism, we conducted metabolomic analysis. SKOV3 ovarian cancer cells were treated with SK4 for 24 h, and then, metabolites were extracted and analysed through mass spectrometry. Quality control was performed on the data with principal component analysis (PCA) and hierarchical clustering analysis (HCA) prior to further analysis (figure). We detected 92 metabolite species. Partial Least-Squares Discriminant Analysis (PLS-DA) was used to detect lipids which underwent the most significant changes. As shown by the heatmap in Figure 3, there were two major clusters of metabolites: a cluster of downregulated metabolites and a cluster of upregulated metabolites. Within the top 25 most significantly perturbed metabolites, 23 metabolites were upregulated (e.g., glucose 1-phosphate, adenosine 5′monophosphate (AMP)) in response to SK4 treatment and 2 were downregulated (see Figure 3, Table S4). This confirms a potential role in energy metabolism. Additionally, substrates of the amino acid transporter LAT1 (the route of entry of SK4) were upregulated including isoleucine, L-phenylalanine and L-valine. This suggests that SK4 may be effluxing out LAT1 allowing entry of the amino acid substrates.

### 3.4. SK4 Reduces Maximal Mitochondrial Respiration and ATP Linked Respiration in SKOV3 and MDA MB 231 Cells

**Figure 4:**
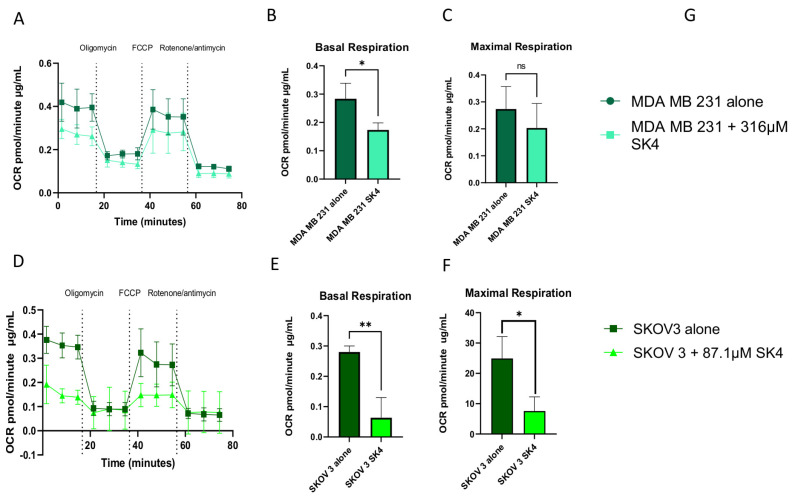
(**A**) Representative graph of the seahorse bioenergetic mito stress test (Agilent Biosciences, Santa Clara, CA, USA) using SK4 pre-treatment for 24 h in MDA MB 231 cells expressed as Oxygen Consumption rate (OCR pmol/min against time). (**B**,**C**) Extrapolated data from graph A of the Mito stress test of SK4 against MDA MB 231 cells, showing basal respiration and maximal respiration. (**D**) Representative graph of the Seahorse bioenergetic mito stress test of SK4 pre-treatment for 24 h n SKOV3 cells (OCR pmol/min against time). (**E**,**F**) Extrapolated data from Mito stress test of SK4 against MDA MB 231. Evaluation of OCR was conducted using Oligomycin 1.5 µM, FCCP 0.5 µM, rotenone/antimycin 0.5 µM and measured by Seahorse XFp Extracellular Flux Analyser (Agilent Technologies, Santa Clara, CA, USA). Each seahorse experiment had been performed in triplicate and one of the representative graphs is shown and all graphs were normalized with total protein using the BCA protein assay following RIPA buffer extraction (**G**). Mean ± SEM calculated from three technical replicates. Statistical analysis using a two-tailed Student’s *t*-test, *p* values below *p* < 0.05 (*) and *p* < 0.01 (**) were established as significant.

Evaluation of the oxygen consumption rate, using Seahorse XFp Extracellular Flux Analyser (Agilent Technologies, Santa Clara, CA, USA), showed that after incubation with SK4 for 24 h there was a significant decrease in the basal mitochondrial respiration of both SKOV3 and MDA MB 231 cells (Figure 4). ATP-linked respiration was determined by the addition of oligomycin and the gap between the baseline basal OCR and the OCR after oligomycin inhibition. The addition of SK4 to the cell for 24 h prior to measuring the mitochondrial respiration resulted in a significant decrease in ATP-linked respiration in both SKOV3 and MDA MB 231 cells, highlighting that SK4 pre-treatment leads to lower production of mitochondrial ATP (Supplementary Figure S1). Furthermore, the addition of FCCP in the Seahorse XFp Mitostress assay allows the measurement of mitochondrial maximum rate of respiration that the cell can achieve. In both cell lines, SK4 exposure for 24 h prior to analysis produced a reduction in the maximal respiration, with SKOV 3 cells achieving a statistically significant reduction in maximal respiration. Furthermore, because basal respiration, observed by the OCR remaining in cells after oligomycin addition (Figure 4 panel A), has not increased in either cell line, there is no evidence of a compensatory increase in glycolysis in SK4 treated cells within 24 h.

### 3.5. SK4 Downregulates Phospholipids in the SKOV3 Cell Lines

**Figure 5:**
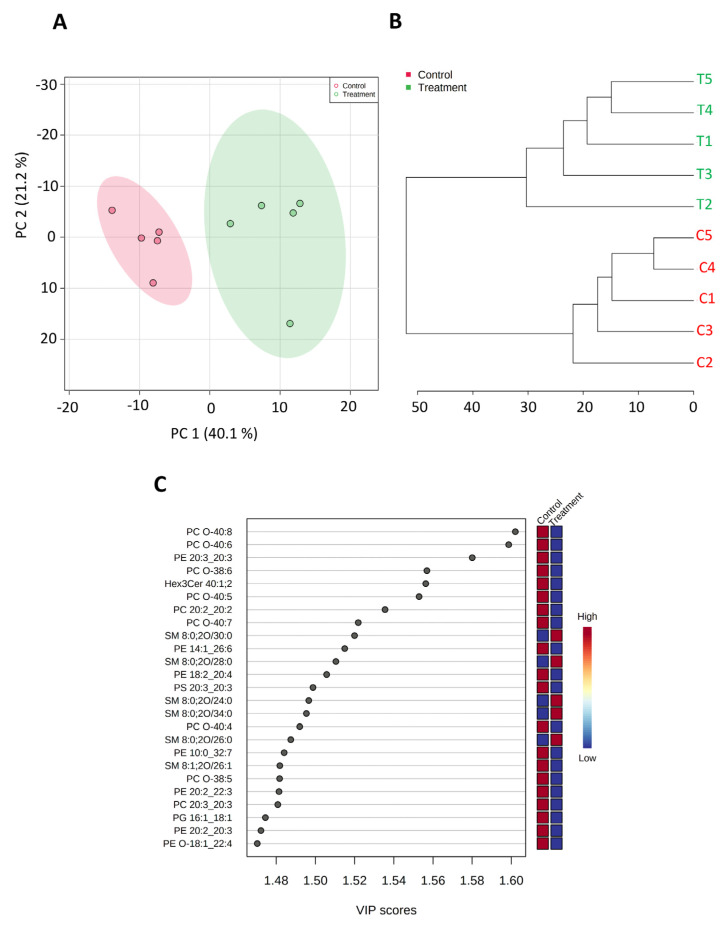
SK4 downregulates phospholipids but upregulates sphingomyelin lipid species in the SKOV3 cells. The lipidomic changes in SKOV3 cells treated with SK4 for 24 h. Quality control of lipidomic data was conducted through PCA (**A**) and HCA (**B**) analysis. Data are representative of 5 biological replicates. (**C**) dot plot of top 25 perturbed lipids VIP score >1 generated through PLS-DA.

In a previously published paper, ceramide induction was observed to drive SK4 mediated apoptosis in the melanoma cell line A375 [15]. SKOV3 cells do not undergo apoptosis when treated with SK4 for 24, 48 or 72 h. To investigate if differences in lipid induction could explain differential responses between different cell lines, lipidomics was utilised on SKOV3 cells treated with SK4 for 24 h. The data underwent quality control through PCA and HCA. Two major clusters were observed in the dot plot of the top 25 lipids with 18 lipid species downregulated and 7 upregulated. Interestingly, phospholipids such as phosphotidylethanolamine (PE), phosphotidylcholine (PC) and phosphotidylserine (PS) (see Figure 5, Table S4), and PE and PS are precursors of the PC synthesis pathway and were observed to be upregulated in previous work with the A375 cells [15]. Although we observed an upregulation of sphingomyelins—the precursor of ceramides—we did not observe an increase in ceramides in the top 25 perturbed lipids.

## 4. Discussion

Overall, the data show energy metabolism is downregulated by SK4 treatment in both the breast cancer MDA MB 231 and ovarian cancer SKOV3 cell lines. Iron deprivation can lead to abnormal mitochondrial biogenesis with dysfunctional cristae [16,17]. Iron deficiency can inhibit the expression of mitochondrial genes at the post transcriptional level through the IRP1/IRP2 proteins. In iron deficient conditions, IRP1 prevents the translation of Bcl-xL inducing mitophagy [18]. Additionally, iron chelation can inhibit the gene expression of mitochondrial proteins (*Sdha*, *Cycs*, *Atp5a1*, *Uqcrc2*, *Ndufa9*, *Ndufs8*) epigenetically through downregulating histone acetylation at H3K9ac [19].

Interestingly, energy metabolism inhibition does not lead to the same phenotypic outcome; in our previous study, only the MDA MB 231 cell line underwent apoptosis but not the SKOV3 cell line [11]. This could indicate differential reliance on mitochondrial metabolism in each cell line, and potentially, that the MDA MB 231 cell line could be more reliant on mitochondrial metabolism; however, this would need further testing to confirm. The iron chelator deferoxamine (DFO) has been shown to inhibit the TCA cycle; however, this resulted in a shift to glycolysis, which may promote resistance over time [20]. We have also shown in real-time measurement assays that SK4 lowers basal and maximal respiration and decreases ATP-coupled respiration. Therefore, SK4 has an inhibitory effect on mitochondrial metabolism. As a measure of energy metabolism impairment, mTOR activity could be assessed to confirm our findings with SK4.

Metabolomics demonstrated an increase in glucose-1-phosphate which could mark an initiation of the shift to glycolysis in the SKOV3 cells. Iron chelation has been shown to upregulate hif-1α which would upregulate GLUT transporter gene expression [21]. This supports a potential case for combining glycolysis inhibitors with iron chelators. Moreover, an upregulation of LAT1 amino acid substrates was observed. LAT1 is an antiport so if there is an increase in intracellular amino acids it may be due to an efflux in SK4, which presents as a limitation towards utilisation of LAT1 as a route of drug entry [22]. LAT1 has been suggested to be a promising target for cancer therapy owing to its relatively higher levels of expression in tumour versus the normal paired tissue [23]. LAT1 overexpressing tumours tend to be more malignant and capable of metastasizing [24]. Previous work showed SK4 can compete with L-phenylalanine (LAT1 substrate) for LAT1 entry; however, this does not negate the potential efflux of SK4 over time [25].

In addition to energy metabolism aberrations, SK4 has also produced significant changes in the lipidome in the SKOV3 cell line. Our data showed a lack of ceramide induction in response to SK4 treatment which contrasts with previous data in the A375 cells [15]. The A375 cells underwent apoptosis, but the SKOV3 cell did not, which could indicate significant ceramide induction is essential for SK4 mediated apoptosis; however, this would need further investigation with more cell lines for confirmation as it is not sufficient to predict the response of other cell lines.

In summary, we have used multiple omics techniques to elucidate the mechanism of action behind SK4 cytotoxicity. Our data demonstrate mitochondrial metabolism as a potential target for SK4 in the MDA MB 231 and SKOV3 cells. However, this may lead to a shift in utilisation of glycolysis as a potential resistance mechanism which would need to be investigated further. Potentially, SK4 could be combined with glycolysis inhibitors. Additionally, our lipidomics work demonstrates differential responses in the SKOV3 ovarian cancer compared to the melanoma cell line A375 with SKOV3 displaying a downregulation in the ceramide induction pathway.

## Data Availability

Proteomics, metabolomics and lipidomics data available in the supplementary materials as Table S4–S6.

## Supplementary Materials

The following supporting information can be downloaded at: https://www.mdpi.com/article/10.3390/biomedicines11072073/s1, Figure S1. (A) Representative graph of the seahorse bioenergetic mito stress test (Agilent Biosciences) using SK4 at 24 h in MDA MB 231 cells expressed as Oxygen Consumption rate shows the ATP Coupled respiration (OCR pmol/minute against time). (B) Representative graph of the Seahorse bioenergetic mito stress test of SK4 at 24 h in SKOV3 cells showing the extrapolated data of ATP coupled respiration (OCR pmol/minute against time). Evaluation of OCR was conducted using Oligomycin 1.5 µM, FCCP 0.5 µM, rotenone/antimycin 0.5 µM and measured by Seahorse XFp Extracellular Flux Analyser (Agilent Technologies. Each seahorse experiment had been performed in triplicate and one of the representative graphs is shown and all graphs were normalized with total protein using the BCA protein assay following RIPA buffer extraction. Mean ±SEM calculated from 3 technical replicates. Statistical analysis using a two tailed Student’s t-test P values below *p* < 0.05 (*) and *p* < 0.01(**) were established as significant. Table S1: Differential expressed proteins in MDA MB 231 cells treated with SK4. Table S2: Differential expressed proteins in SKOV3 cells treated with SK4. Table S3: 54 commonly differentially expressed proteins in the MDA MB 231 and SKOV3 cells. Table S4: Metabolomics and lipidomic cell profile. Table S5: LFQ values of MDA MB 231 cells treated with SK4 for 24 h. Table S6: LFQ values of SKOV3 cells treated with SK4 for 24 h.

## References

[1] Gutierrez E., Richardson D.R., Jansson P.J. (2014). The anticancer agent di-2-pyridylketone 4,4-dimethyl-3-thiosemicarbazone (Dp44mT) overcomes prosurvival autophagy by two mechanisms: Persistent induction of autophagosome synthesis and impairment of lysosomal integrity. J. Biol. Chem..

[2] Yu Y., Suryo Rahmanto Y., Hawkins C.L., Richardson D.R. (2011). The potent and novel thiosemicarbazone chelators di-2-pyridylketone-4,4-dimethyl-3-thiosemicarbazone and 2-benzoylpyridine-4,4-dimethyl-3-thiosemicarbazone affect crucial thiol systems required for ribonucleotide reductase activity. Mol. Pharmacol..

[3] Harima H., Kaino S., Takami T., Shinoda S., Matsumoto T., Fujisawa K., Yamamoto N., Yamasaki T., Sakaida I. (2016). Deferasirox, a novel oral iron chelator, shows antiproliferative activity against pancreatic cancer in vitro and in vivo. BMC Cancer.

[4] Chen Z., Zhang D., Yue F., Zheng M., Kovacevic Z., Richardson D.R. (2012). The iron chelators Dp44mT and DFO inhibit TGF-beta-induced epithelial-mesenchymal transition via up-regulation of N-Myc downstream-regulated gene 1 (NDRG1). J. Biol. Chem..

[5] Lui G.Y., Kovacevic Z., S V.M., Kalinowski D.S., Merlot A.M., Sahni S., Richardson D.R. (2015). Novel thiosemicarbazones regulate the signal transducer and activator of transcription 3 (STAT3) pathway: Inhibition of constitutive and interleukin 6-induced activation by iron depletion. Mol. Pharmacol..

[6] Kovacevic Z., Chikhani S., Lui G.Y., Sivagurunathan S., Richardson D.R. (2013). The iron-regulated metastasis suppressor NDRG1 targets NEDD4L, PTEN, and SMAD4 and inhibits the PI3K and Ras signaling pathways. Antioxid. Redox Signal.

[7] Lin Z.P., Zhu Y.L., Lo Y.C., Moscarelli J., Xiong A., Korayem Y., Huang P.H., Giri S., LoRusso P., Ratner E.S. (2018). Combination of triapine, olaparib, and cediranib suppresses progression of BRCA-wild type and PARP inhibitor-resistant epithelial ovarian cancer. PLoS ONE.

[8] Finch R.A., Liu M., Grill S.P., Rose W.C., Loomis R., Vasquez K.M., Cheng Y., Sartorelli A.C. (2000). Triapine (3-aminopyridine-2-carboxaldehyde- thiosemicarbazone): A potent inhibitor of ribonucleotide reductase activity with broad spectrum antitumor activity. Biochem. Pharmacol..

[9] Attia S., Kolesar J., Mahoney M.R., Pitot H.C., Laheru D., Heun J., Huang W., Eickhoff J., Erlichman C., Holen K.D. (2008). A phase 2 consortium (P2C) trial of 3-aminopyridine-2-carboxaldehyde thiosemicarbazone (3-AP) for advanced adenocarcinoma of the pancreas. Investig. New Drugs.

[10] Kyriakou S., Mitsiogianni M., Mantso T., Cheung W., Todryk S., Veuger S., Pappa A., Tetard D., Panayiotidis M.I. (2020). Anticancer activity of a novel methylated analogue of L-mimosine against an in vitro model of human malignant melanoma. Investig. New Drugs.

[11] Abdelaal G., Carter A., Panayiotides M.I., Tetard D., Veuger S. (2022). Novel iron chelator SK4 demonstrates cytotoxicity in a range of tumour derived cell lines. Front. Mol. Biosci..

[12] Tyanova S., Temu T., Sinitcyn P., Carlson A., Hein M.Y., Geiger T., Mann M., Cox J. (2016). The Perseus computational platform for comprehensive analysis of (prote)omics data. Nat. Methods.

[13] Ritchie M.E., Phipson B., Wu D., Hu Y., Law C.W., Shi W., Smyth G.K. (2015). limma powers differential expression analyses for RNA-sequencing and microarray studies. Nucleic Acids Res..

[14] Yu G., He Q.Y. (2016). ReactomePA: An R/Bioconductor package for reactome pathway analysis and visualization. Mol. Biosyst..

[15] Kyriakou S., Cheung W., Mantso T., Mitsiogianni M., Anestopoulos I., Veuger S., Trafalis D.T., Franco R., Pappa A., Tetard D. (2021). A novel methylated analogue of L-Mimosine exerts its therapeutic potency through ROS production and ceramide-induced apoptosis in malignant melanoma. Investig. New Drugs.

[16] Masini A., Salvioli G., Cremonesi P., Botti B., Gallesi D., Ceccarelli D. (1994). Dietary iron deficiency in the rat. I. Abnormalities in energy metabolism of the hepatic tissue. Biochim. Biophys. Acta.

[17] Jarvis J.H., Jacobs A. (1974). Morphological abnormalities in lymphocyte mitochondria associated with iron-deficiency anaemia. J. Clin. Pathol..

[18] Wu H., Wei H., Zhang D., Sehgal S.A., Zhang D., Wang X., Qin Y., Liu L., Chen Q. (2020). Defective mitochondrial ISCs biogenesis switches on IRP1 to fine tune selective mitophagy. Redox Biol..

[19] Rensvold J.W., Krautkramer K.A., Dowell J.A., Denu J.M., Pagliarini D.J. (2016). Iron Deprivation Induces Transcriptional Regulation of Mitochondrial Biogenesis. J. Biol. Chem..

[20] Oexle H., Gnaiger E., Weiss G. (1999). Iron-dependent changes in cellular energy metabolism: Influence on citric acid cycle and oxidative phosphorylation. Biochim. Biophys. Acta.

[21] Torti S.V., Torti F.M. (2013). Iron and cancer: More ore to be mined. Nat. Rev. Cancer.

[22] Meier C., Ristic Z., Klauser S., Verrey F. (2002). Activation of system L heterodimeric amino acid exchangers by intracellular substrates. EMBO J..

[23] Kurozumi S., Kaira K., Matsumoto H., Kurosumi M., Yokobori T., Kanai Y., Sekine C., Honda C., Katayama A., Furuya M. (2022). Association of L-type amino acid transporter 1 (LAT1) with the immune system and prognosis in invasive breast cancer. Sci. Rep..

[24] Ichinoe M., Yanagisawa N., Mikami T., Hana K., Nakada N., Endou H., Okayasu I., Murakumo Y. (2015). L-Type amino acid transporter 1 (LAT1) expression in lymph node metastasis of gastric carcinoma: Its correlation with size of metastatic lesion and Ki-67 labeling. Pathol. Res. Pract..

[25] Gutbier S., Kyriakou S., Schildknecht S., Uckert A.K., Brull M., Lewis F., Dickens D., Pearson L., Elson J.L., Michel S. (2020). Design and evaluation of bi-functional iron chelators for protection of dopaminergic neurons from toxicants. Arch. Toxicol..

