# Supplementary material for: Novel Iron Chelator SK4 Drives Cytotoxicity through Inhibiting Mitochondrial Metabolism in Ovarian and Triple Negative Breast Cancer Cell Lines
Source: Biomedicines. 2023 Jul 24;11(7):2073. doi: 10.3390/biomedicines11072073 (PMC10377004; doi:10.3390/biomedicines11072073)
Supplement: Supplementary file 1 [file biomedicines-11-02073-s001.zip › Supplementary figure S1 .pdf]

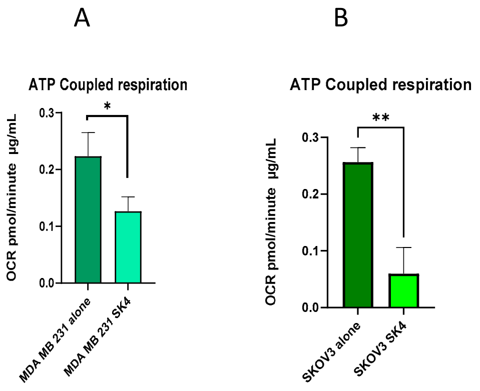

Supplementary Figure S1. (A) Representative graph of the seahorse bioenergetic mito stress test (Agilent Biosciences) using SK4 at 24 hours in MDA MB 231 cells expressed as Oxygen Consumption rate shows the ATP Coupled respiration (OCR pmol/minute against time). (B) Representative graph of the Seahorse bioenergetic mito stress test of SK4 at 24 hours in SKOV3 cells showing the extrapolated data of ATP coupled respiration (OCR pmol/minute against time). Evaluation of OCR was conducted using Oligomycin 1.5µM, FCCP 0.5µM, rotenone/antimycin 0.5µM and measured by Seahorse XFp Extracellular Flux Analyser (Agilent Technologies. Each seahorse experiment had been performed in triplicate and one of the representative graphs is shown and all graphs were normalized with total protein using the BCA protein assay following RIPA buffer extraction. Mean ±SEM calculated from 3 technical replicates. Statistical analysis using a two tailed Student's t-test P values below p<0.05(*) and p<0.01(**) were established as significant.
